# Disordered Eating Behaviors Among U.S. Children With Neuropsychiatric Conditions: A Nationally Representative Study

**DOI:** 10.1002/eat.70100

**Published:** 2026-04-12

**Authors:** Myriam Casseus

**Affiliations:** ^1^ Division of Population Health, Quality, and Implementation Sciences, Department of Pediatrics, Robert Wood Johnson Medical School Rutgers University New Brunswick New Jersey USA; ^2^ Child Health Institute of New Jersey New Brunswick New Jersey USA

**Keywords:** anxiety, attention‐deficit/hyperactivity disorder, depression, disordered eating, eating behaviors, neuropsychiatric conditions and disordered eating

## Abstract

**Objective:**

This study documented associations between parent‐reported disordered eating behaviors and co‐occurring anxiety, depression, behavioral/conduct problems, and attention‐deficit/hyperactivity disorder (ADHD) among U.S. children.

**Method:**

Data were obtained from the combined 2022–2023 National Survey of Children's Health. The sample consisted of 68,000 children aged 6–17 years. Prevalence was estimated for sociodemographic variables, neuropsychiatric disorders, and the following disordered eating behaviors: (1) skipping meals or fasting; (2) having low interest in food; (3) extremely picky eating; (4) binge eating; (5) purging or vomiting; (6) using diet pills, laxatives, or diuretics; (7) over‐exercising; and (8) not eating due to fear of vomiting or choking. Multiple logistic regression analysis assessed associations between neuropsychiatric disorders and disordered eating behaviors, adjusting for sociodemographic factors.

**Results:**

Prevalence of any disordered eating behavior was 30.1% (95% CI, 29.4%–30.8%). Higher odds of disordered eating behaviors were associated with anxiety (aOR [adjusted odds ratio]: 3.64, 95% CI, 3.34–3.96), depression (aOR: 4.59, 95% CI, 3.99–5.28), behavioral/conduct problems (aOR: 3.40, 95% CI, 3.03–3.81), and ADHD (aOR: 2.63, 95% CI, 2.41–2.88). Significant associations were observed across nearly all examined eating behaviors.

**Discussion:**

This is the first nationally representative study to find that U.S. children and adolescents with neuropsychiatric disorders were more likely to exhibit disordered eating behaviors than their counterparts without neuropsychiatric disorders. Findings underscore the need for screening and early intervention to optimize the mental and physical health of these populations. They also highlight the need to address prevention and treatment of these disorders in the context of psychiatric comorbidities.

## Summary

- This nationally representative study found that children with anxiety, depression, behavioral/conduct problems, and attention‐deficit/hyperactivity disorder were more likely to exhibit disordered eating behaviors than children without the disorders.
- With the high rate of morbidity and mortality associated with eating disorders, findings underscore the need for screening and early intervention to optimize the mental and physical health of children with these disorders.
- Findings also highlight the need to address prevention and treatment of these disorders in the context of psychiatric comorbidities.

## 1 Introduction

Disordered eating is characterized by severe and persistent disturbance in eating or weight control behaviors and affects between 5.2% and 22.4% of children and adolescents globally (American Psychiatric Association 2013; Faria et al. 2026; López‐Gil et al. 2023). Prevalence of disordered eating among U.S. children and adolescents ranges from 5.6% to 22.5% (López‐Gil et al. 2023). These disorders can lead to serious physical health problems and are associated with bone fractures, anemia, osteoporosis, malnutrition, electrolyte imbalance, dental erosion, obesity, diabetes, hypertension, high cholesterol, and high triglycerides (Attia and Walsh 2025; Faje et al. 2014; Udo and Grilo 2019). Eating disorders also have among the highest mortality rates of any psychiatric illness (Krug et al. 2025; Semchishen et al. 2026).

Eating disorders are highly comorbid with other psychiatric disorders (Convertino and Blashill 2022; Sanzari et al. 2023; Tsai et al. 2018). The specific mechanisms undergirding this comorbid relationship are not well understood and appear to be a multifaceted combination of genetic, epigenetic, neurobiological, psychological, developmental, sociocultural, familial, and environmental factors (Hambleton et al. 2022; Momen et al. 2022; Sanzari et al. 2023). There is evidence of potential genetic links between disordered eating and anxiety (Hambleton et al. 2022). Emotional dysregulation is associated with impulsive eating disorder behaviors and attention‐deficit/hyperactivity disorder (ADHD) (Reinblatt 2015) and is also common in the psychopathology of eating disorder and eating‐related symptoms (Lavender et al. 2015; Prefit et al. 2019).

Several studies that include adolescents show high rates of psychiatric disorders among individuals with eating disorders, namely anxiety, mood disorders, obsessive‐compulsive disorder (OCD), ADHD, and post‐traumatic stress disorder (Convertino and Blashill 2022; Katzman et al. 2022; Sanzari et al. 2023; Tsai et al. 2018). Prevalences of these comorbidities among adolescents are difficult to assess as these studies often combine findings for children, adolescents, and adults (Katzman et al. 2022; Lin et al. 2024; Tsai et al. 2018). Other limitations of these studies include small sample sizes and results from mostly clinically referred samples (Fisher et al. 2014; Katzman et al. 2022). An older study using data from adolescents in a small community sample found that adolescents with eating disorders were more likely to have panic disorders or mood disorders, and that OCD was predictive of eating disorders (Zaider et al. 2000). Little is known about comorbid neuropsychiatric disorders among children younger than 12 years old with disordered eating behaviors (Sanchez‐Cerezo et al. 2023). Two notable exceptions are studies that found that anxiety disorders, ADHD, disruptive/impulse control disorders, mood disorders, and OCD were more prevalent among children aged 9–10 years diagnosed with eating disorders compared with their counterparts without the disorders (Convertino and Blashill 2022; Sanzari et al. 2023).

Disordered eating behaviors include restrictive eating; emotional eating; disinhibited eating; night eating; binge eating; strict dieting; and controlling one's body weight and shape through compensatory behaviors, such as self‐induced vomiting (purging), excessive exercise, and the use of laxatives or diuretics (Beccia et al. 2025; López‐Gil et al. 2023). Many individuals who exhibit these behaviors do not meet diagnostic criteria for an eating disorder, yet these behaviors may cause serious physical and psychological harm and lead to the development of diagnosed eating disorders in later years (Neumark‐Sztainer et al. 2006; Schaumberg and Anderson 2016).

There is currently no research estimating associations between disordered eating behaviors and co‐occurring neuropsychiatric disorders in a large U.S. nationally representative sample. It is important to use public health surveillance tools to systematically identify and monitor these behaviors to inform prevention efforts and evidence‐based interventions. Therefore, this study documented associations between disordered eating behaviors and co‐occurring anxiety, depression, behavioral/conduct problems, and ADHD among U.S. children and adolescents aged 6–17 years.

## 2 Method

### 2.1 Data

Data were obtained from the combined 2022–2023 National Survey of Children's Health (NSCH), a serial cross‐sectional survey of noninstitutionalized U.S. children aged 0 through 17 years (U.S. Census Bureau 2025a). The NSCH was designed to produce nationally‐ and state‐representative measures related to the health of children, including physical and mental health, access to quality health care, family, neighborhood, school, and social factors (Data Resource Center for Child and Adolescent Health 2023a). The survey used stratification and address‐based sampling of the 50 states and the District of Columbia. Addresses were randomly sampled within states. Detailed information on NSCH methodology is available from the Data Resource Center for Child and Adolescent Health (2023b).

Respondents were parents or guardians (hereafter, “parents”) of a randomly selected child in a sampled household. Overall survey response rates were 39.1% (2022) and 35.8% (2023) (U.S. Census Bureau 2025b). The combined NSCH sample included 109,265 children and adolescents, 68,000 of whom were aged 6–17 years. Based on the U.S. Census, survey weights were applied to account for unequal probabilities of selection and survey nonresponse, and to reflect the population of children aged 0–17 years. All NSCH public use data files went through a disclosure review process and were approved by the Census Disclosure Review Board prior to release (Data Resource Center for Child and Adolescent Health 2023b).

### 2.2 Measures

To ascertain neuropsychiatric conditions, parents affirmed that a doctor or other health care provider had told them that their child currently has anxiety, depression, or ADHD. For behavioral/conduct problems, parents were asked whether a doctor, other health care provider, or educator had told them that their child had behavioral/conduct problems (examples of educators are teachers and school nurses). Although this question has the potential to result in overreporting or underreporting of ADHD by parents, studies have shown convergent validity and reliability of the NSCH parent‐reported ADHD diagnosis and Diagnostic and Statistical Manual criteria for ADHD (Cree et al. 2023; Visser et al. 2013). For disordered eating behaviors, parents were asked whether the child engaged in any of the following behaviors during the past 12 months: (1) skipping meals or fasting; (2) having low interest in food; (3) extremely picky eating; (4) binge eating; (5) purging or vomiting after eating; (6) using diet pills, laxatives, or diuretics to lose or maintain weight without a doctor's orders; (7) over‐exercising; and (8) not eating due to fear of vomiting or choking. These questions have been used previously to measure eating‐ or body image‐related behaviors among children and adolescents (Lu 2025).

### 2.3 Covariates

Potential confounders were informed by previous research and included child demographic variables: gender, age, race and ethnicity, and health insurance status. Household variables included poverty (based on the federal poverty level (FPL) guidelines and self‐reported household annual income), household educational attainment, and household language (Casseus and Reichman 2025; Convertino and Blashill 2022; Fisher et al. 2014; Hahn et al. 2025; Hornberger et al. 2021; Lu 2025).

### 2.4 Statistical Analyses

Prevalence and 95% confidence intervals were calculated for sociodemographic variables (sex, age, race and ethnicity, household poverty, household educational attainment, health insurance status, and household language), neuropsychiatric disorders and disordered eating behaviors. Multiple logistic regression analysis assessed associations between neuropsychiatric disorders and disordered eating behaviors adjusted for sociodemographic variables. Adjusted odds ratios (aORs) and 95% confidence intervals (CIs) were reported. Analyses were weighted to account for the complex sample design, probability of selection, and nonresponse. The NSCH used hot‐deck and regression methods to impute missing demographic variables (U.S. Census Bureau 2025a). Listwise deletion was used for missing mental health and disordered eating behaviors variables (< 1%). Analyses were conducted using SAS statistical software version 9.4 (SAS Institute, Cary, NC).

## 3 Results

An estimated 12.7% (95% CI, 12.3%–13.1%) of children and adolescents had anxiety, 5.4% (95% CI, 5.1%–5.7%) had depression, 8.1% (95% CI, 7.7%–8.5%) had behavioral/conduct problems, and 12.4 (95% CI, 12.0%–12.9%) had ADHD (Tables 1 and S1). Prevalence of any disordered eating behavior was 30.1% (95% CI, 29.4%–30.8%), with extremely picky eating (21.2; 95% CI, 20.6%–21.8%) emerging as the most common disordered eating behavior, followed by low interest in food (10.4; 95% CI, 10.0%–10.9%), skipping meals or fasting (10.0%; 95% CI, 9.6%–10.4%), and binge eating (4.8%; 95% CI, 4.4%–5.1%).

**TABLE 1:** Sample characteristics of children aged 6–17 years.

Characteristics	Unweighted *n* = 68,000	Weighted % (95% CI)
Sex		
Male	35,270	51.2 (50.5–52.0)
Female	32,730	48.8 (48.3–49.5)
Age (years)		
6–9	20,383	31.8 (31.1–32.5)
10–12	15,377	24.9 (24.2–25.6)
13–17	32,240	43.3 (42.6–44.0)
Race/ethnicity		
Asian non‐Hispanic	4368	5.0 (4.8–5.3)
Black non‐Hispanic	4498	13.2 (12.6–13.8)
Hispanic	10,502	27.2 (26.4–27.9)
Multi‐racial/other non‐Hispanic	5211	7.3 (7.0–7.6)
White non‐Hispanic	43,421	47.3 (46.5–48.0)
Household poverty		
< 100% FPL	8866	18.3 (17.6–18.9)
100%–199% FPL	11,206	19.8 (19.1–20.4)
200%–399% FPL	19,925	29.0 (28.4–29.7)
≥ 400% FPL	28,003	32.9 (32.3–33.5)
Household educational attainment		
Less than high school	2016	9.3 (8.7–9.9)
High school/GED	9282	19.2 (18.6–19.9)
Some college or technical school	14,693	20.0 (19.4–20.5)
College degree or higher	42,009	51.5 (50.8–52.3)
Insurance status		
Private only	45,421	58.3 (57.5–59.3)
Public only	14,949	29.4 (28.6–30.1)
Public and private	3256	5.3 (5.0–5.7)
Currently uninsured	3008	7.0 (6.5–7.5)
Language spoken at home		
English	61,895	84.6 (83.9–85.3)
Other than English	5692	15.4 (14.7–16.1)
Neuropsychiatric conditions		
Anxiety	10,968	12.7 (12.3–13.1)
Depression	4749	5.4 (5.1–5.7)
Behavioral/conduct problems	5981	8.1 (7.7–8.5)
ADHD	9795	12.4 (12.0–12.9)
Disordered eating behaviors		
Any disordered eating behavior	21,136	30.1 (29.4–30.8)
Extremely picky eating	14,953	21.2 (20.6–21.8)
Low interest in food	7760	10.4 (10.0–10.9)
Skipping meals or fasting	7538	10.0 (9.6–10.4)
Binge eating	3163	4.8 (4.4–5.1)
Over‐exercising	718	1.1 (0.9–1.2)
Fear of vomiting or choking	664	0.8 (0.7–0.9)
Purging or vomiting	431	0.7 (0.5–0.8)
Using diet pills, laxatives, or diuretics	102	0.1 (0.1–0.2)

Abbreviations: ADHD, attention‐deficit/hyperactivity disorder; CI, confidence interval; FPL, federal poverty level (the ratio of total family income to the family poverty threshold); GED, General Equivalency Diploma.

*Source:* National Survey of Children's Health.

Anxiety was associated with higher odds of disordered eating behaviors (aOR: 3.64, 95% CI, 3.34–3.96) and all of the disordered eating behaviors examined, including fear of vomiting or choking (aOR: 6.77, 95% CI, 5.03–9.11) and purging or vomiting (aOR: 4.73, 95% CI, 3.24–6.89) (Table 2 and Supplemental Tables S2–S5). Similarly, children with depression had higher odds of disordered eating behaviors (aOR: 4.59, 95% CI, 3.99–5.28) and all of the disordered eating behaviors, particularly fear of vomiting or choking (aOR: 6.31, 95% CI, 4.52–8.81) and purging or vomiting (aOR: 6.26, 95% CI, 4.29–9.14). Behavioral/conduct problems were associated with higher odds of disordered eating behaviors (aOR: 3.40, 95% CI, 3.03–3.81) and all listed disordered eating behaviors. Similarly, ADHD was associated with higher odds of disordered eating behaviors (aOR: 2.63, 95% CI, 2.41–2.88) and all listed eating behaviors except for using diet pills, laxatives, or diuretics.

**TABLE 2:** Disordered eating behaviors among children aged 6–17 years with neuropsychiatric comorbidities.

Disordered eating behaviors	Anxiety	Depression	Behavioral/conduct problems	ADHD
	aOR (95% CI)	aOR (95% CI)	aOR (95% CI)	aOR (95% CI)
Any disordered eating behavior	3.64 (3.34–3.96)***	4.59 (3.99–5.28)***	3.40 (3.03–3.81)***	2.63 (2.41–2.88)***
Extremely picky eating	3.06 (2.80–3.34)***	2.98 (2.62–3.38)***	2.88 (2.57–3.22)***	2.37 (2.16–2.59)***
Low interest in food	4.20 (3.79–4.66)***	5.61 (4.90–6.42)***	3.47 (3.03–3.97)***	3.03 (2.70–3.39)***
Skipping meals or fasting	3.50 (3.16–3.87)***	4.38 (3.83–5.02)***	2.79 (2.44–3.19)***	2.44 (2.19–2.72)***
Binge eating	4.29 (3.68–5.00)***	5.38 (4.50–6.45)***	5.04 (4.25–5.99)***	3.26 (2.79–3.80)***
Over‐exercising	2.42 (1.80–3.26)***	3.38 (2.39–4.77)***	1.94 (1.25–3.02)**	1.60 (1.07–2.38)*
Fear of vomiting or choking	6.77 (5.03–9.11)***	6.31 (4.52–8.81)***	2.56 (1.77–3.72)***	2.12 (1.50–2.98)***
Purging or vomiting	4.73 (3.24–6.89)***	6.26 (4.29–9.14)***	4.00 (2.60–6.15)***	2.74 (1.81–4.16)***
Using diet pills, laxatives, or diuretics	2.96 (1.45–6.04)**	4.56 (2.12–9.77)***	2.81 (1.04–7.59)*	1.84 (0.80–4.20)

*Note:* Models adjusted for sex, age, race/ethnicity, household poverty, household educational attainment, insurance status, and household language.

Abbreviations: ADHD, attention‐deficit/hyperactivity disorder; aOR, adjusted odds ratio; CI, confident interval.

* *p* < 0.05.

** *p* < 0.01.

*** *p* < 0.001.

*Source:* National Survey of Children's Health.

## 4 Discussion

Research on the co‐occurrence of neuropsychiatric conditions and disordered eating behaviors among young children and adolescents is necessary as the rates of these disorders have increased in the past decade (Casseus and Reichman 2025; Pastore et al. 2023). This study examined the associations between co‐occurring disordered eating behaviors and anxiety, depression, behavioral/conduct problems, and ADHD among a large nationally representative sample of U.S. children. Findings suggest that children with these neuropsychiatric disorders are more likely to exhibit disordered eating behaviors.

Consistent with previous research, this study found that disordered eating was highly comorbid with neuropsychiatric disorders (Katzman et al. 2022; Sanchez‐Cerezo et al. 2023). After adjusting for sociodemographic factors to avoid confounding of results, this study found that compared to children without neuropsychiatric disorders, children diagnosed with depression had more than four times higher odds of any of the disordered eating behaviors examined. Individuals with anxiety or behavioral/conduct problems were three times more likely to exhibit disordered eating behaviors, while those with ADHD had greater than twice the odds of disordered eating.

Significant associations were observed across nearly all eating behaviors examined. Notably, purging or vomiting and using diet pills, laxatives, or diuretics were highly prevalent among children with neuropsychiatric disorders. These behaviors can lead to serious complications including dehydration, loss of electrolytes, and bradycardia (Forney et al. 2016; Milliren et al. 2022). Other complications caused by these behaviors include stomach and esophageal problems (e.g., gastroesophageal reflux) and gastrointestinal disorders (e.g., irritable bowel syndrome) (Forney et al. 2016). The odds of binge eating were three to five times higher among children with neuropsychiatric disorders. Binge eating is associated with obesity, diabetes, hypertension, high cholesterol, and high triglycerides (Udo and Grilo 2019). With the high rate of morbidity and mortality associated with eating disorders, findings also underscore the need for early intervention to optimize the mental and physical health of children with these disorders.

Emotional dysregulation, self‐esteem deficits, interpersonal problems, and negative affect (i.e., anxiety and depression) are risk factors for disordered eating (Pennesi and Wade 2016). For example, children with ADHD present with executive function deficits (such as attention problems and impulsive behavior) which are associated with obesity and binge‐eating behavior (Reinblatt 2015). Childhood trauma is also implicated with a recent study finding positive associations between adverse childhood experiences and disordered eating behaviors (Lu 2025). These findings point to multiple avenues of future research to understand the pathophysiology and mechanisms associated with disordered eating and neuropsychiatric disorders.

Eating disorders and disordered eating behaviors often go undiagnosed and untreated (Forrest et al. 2017; Hornberger et al. 2021; López‐Gil et al. 2023). Therefore, it is essential for family members, health care professionals, policymakers, and other stakeholders to be aware of behaviors which may signal undiagnosed eating disorders or subthreshold eating disorders. These behaviors may also lead to later eating disorders (Schaumberg and Anderson 2016). Screening for these comorbid disorders in both primary care and pediatric mental health care settings is essential. Furthermore, robust efforts are required to develop effective interventions for disordered eating that incorporate treatment of anxiety, depression, behavioral/conduct problems, ADHD, and other neuropsychiatric disorders.

The limitations of this study include that neuropsychiatric conditions and disordered eating behaviors were parent‐reported, and thus subject to potential reporting biases and not independently verified by a review of medical records. Given the inherent secrecy of certain disordered eating behaviors (e.g., binge eating, purging or vomiting and using diet pills, laxatives, or diuretics) and the shame associated with them (Blythin et al. 2020), parents may not be aware of these behaviors which can lead to underreporting. This is particularly true for adolescents who have more autonomy, independence, and access to weight loss information and products than younger children. Furthermore, parents were asked whether a doctor, other health care provider, or educator had told them that their child had a neuropsychiatric condition. The question did not ask whether the child had been screened or diagnosed with the condition. That said, research has demonstrated the validity of parent reports with regard to the NSCH (Cree et al. 2023; Visser et al. 2013). Additionally, the data were cross‐sectional and did not allow for causal inference.

Nevertheless, this is the first known study to estimate associations between childhood anxiety, depression, behavioral/conduct problems, ADHD, and disordered eating behaviors in a large U.S. nationally representative sample. Findings that children with these neuropsychiatric disorders are more likely to exhibit disordered eating behaviors highlight the need to better address prevention and treatment of these disorders in the context of psychiatric comorbidities.

## Author Contributions

**Myriam Casseus:** conceptualization, investigation, funding acquisition, writing – original draft, methodology, validation, visualization, writing – review and editing, software, formal analysis, project administration, data curation, supervision, resources.

## Lived Experience Involvement Statement

No specific efforts were undertaken to involve persons with lived experience in the study design or execution, or in the preparation of this manuscript.

## Funding

Funding for this study was provided by the Robert E. Leet and Clara Guthrie Patterson Trust. The contents are those of the author and do not necessarily represent the official views of, nor an endorsement by, the sponsor.

## Conflicts of Interest

The author declares no conflicts of interest.

## Supporting information


**Table S1:** Prevalence of co‐occurring neuropsychiatric disorders among children aged 6–17 years.
**Table S2:** Multivariable logistic regression of associations between anxiety and any disordered eating behaviors.
**Table S3:** Multivariable logistic regression of associations between depression and any disordered eating behaviors.
**Table S4:** Multivariable logistic regression of associations between behavioral/conduct problems and any disordered eating behaviors.
**Table S5:** Multivariable logistic regression of associations between attention‐deficit/hyperactivity disorder and any disordered eating behaviors.

## Data Availability

This study utilized publicly available data from https://www.census.gov/programs‐surveys/nsch/data/datasets.html.
