# Supplementary material for: Disordered Eating Behaviors Among U.S. Children With Neuropsychiatric Conditions: A Nationally Representative Study
Source: Int J Eat Disord. 2026 Apr 12;59(8):1853–9. doi: 10.1002/eat.70100 (PMC13446250; doi:10.1002/eat.70100)
Supplement: Supplementary file 1 [file EAT-59-1853-s001.docx]

**Supplemental material**

Table S1. Prevalence of co-occurring neuropsychiatric disorders among children aged 6-17 years.

| **Co-occurring neuropsychiatric disorders** | **Unweighted N** | **Weighted % (CI)** |
| --- | --- | --- |
| Anxiety and depression | 4,000 | 4.3 (4.1-4.5) |
| Anxiety and ADHD | 4,368 | 4.8 (4.6-5.1) |
| Anxiety and behavioral/conduct problems | 2,886 | 3.5 (3.2-3.7) |
| Depression and ADHD | 2,055 | 2.2 (2.1-2.4) |
| Depression and behavioral/conduct problems | 1,438 | 1.7 (1.5-1.9) |
| ADHD and behavioral/conduct problems | 4,052 | 5.3 (4.9-5.6) |
| Anxiety, depression, and ADHD | 1,828 | 1.8 (1.7-2.0) |
| Anxiety, depression, ADHD, and behavioral/conduct problems | 941 | 1.0 (0.9-1.1) |

Source: National Survey of Children’s Health.

Abbreviations: ADHD, Attention-deficit/hyperactivity disorder; CI, confidence interval.

Table S2. Multivariable logistic regression of associations between anxiety and any disordered eating behaviors.

| **Characteristics** | **aOR (95% CI)** |
| --- | --- |
| **Anxiety** | 3.64 (3.34-3.96)*** |
| **Sex** |  |
| Male | REF |
| Female | 1.01 (0.94-1.08) |
| **Age (yrs.)** |  |
| 6-9 | REF |
| 10-12 | 0.89 (0.81-0.98)* |
| 13-17 | 0.82 (0.76-0.89)*** |
| **Race/ethnicity** |  |
| Asian non-Hispanic | 0.88 (0.76-1.02) |
| Black non-Hispanic | 1.25 (1.11-1.41)*** |
| Hispanic | 1.22 (1.11-1.34)*** |
| Multi-racial/Other non-Hispanic | 1.17 (1.05-1.30)** |
| White non-Hispanic | REF |
| **Household poverty** |  |
| < 100% FPL | 0.99 (0.87-1.12) |
| 100-199% FPL | 0.96 (0.86-1.07) |
| 200-399% FPL | 1.08 (1.00-1.17) |
| ≥400% FPL | REF |
| **Household educational attainment** |  |
| Less than high school | 1.07 (0.89-1.30) |
| High school /GED | 1.09 (0.98-1.22) |
| Some college or technical school | 1.05 (0.96-1.14) |
| College degree or higher | REF |
| **Insurance status** |  |
| Private only | REF |
| Public only | 1.29 (1.17-1.43)*** |
| Public and private | 1.59 (1.37-1.86)*** |
| Currently uninsured | 1.11 (0.93-1.33) |
| **Household Language** |  |
| English | REF |
| Other than English | 1.05 (0.91-1.21) |

Source: National Survey of Children’s Health.

Abbreviations: aOR, adjusted odds ratio; CI, confident interval; FPL, federal poverty level (the ratio of total family income to the family poverty threshold); GED, General Equivalency Diploma.

Models adjusted for sex, age, race/ethnicity, household poverty, household educational attainment, insurance status, and household language.

*p<.05, **p<.01, ***p<.001

Table S3. Multivariable logistic regression of associations between depression and any disordered eating behaviors.

| **Characteristics** | **aOR (95% CI)** |
| --- | --- |
| **Depression** | 4.59 (3.99-5.28)*** |
| **Sex** |  |
| Male | REF |
| Female | 1.01 (0.95-1.08) |
| **Age (yrs.)** |  |
| 6-9 | REF |
| 10-12 | 0.92 (0.84-1.00) |
| 13-17 | 0.82 (0.76-0.88)*** |
| **Race/ethnicity** |  |
| Asian non-Hispanic | 0.78 (0.67-0.91)** |
| Black non-Hispanic | 1.14 (1.02-1.28)* |
| Hispanic | 1.18 (1.07-1.30)*** |
| Multi-racial/Other non-Hispanic | 1.13 (1.01-1.25)* |
| White non-Hispanic | REF |
| **Household poverty** |  |
| < 100% FPL | 1.00 (0.88-1.14) |
| 100-199% FPL | 0.95 (0.85-1.06) |
| 200-399% FPL | 1.08 (0.99-1.17) |
| ≥400% FPL | REF |
| **Household educational attainment** |  |
| Less than high school | 0.99 (0.82-1.21) |
| High school/GED | 1.06 (0.96-1.18) |
| Some college or technical school | 1.02 (0.93-1.11) |
| College degree or higher | REF |
| **Insurance status** |  |
| Private only | REF |
| Public only | 1.28 (1.16-1.42)*** |
| Public and private | 1.60 (1.38-1.86)*** |
| Currently uninsured | 1.06 (0.89-1.27) |
| **Household Language** |  |
| English | REF |
| Other than English | 1.06 (0.92-1.23) |

Source: National Survey of Children’s Health.

Abbreviations: aOR, adjusted odds ratio; CI, confident interval; FPL, federal poverty level (the ratio of total family income to the family poverty threshold); GED, General Equivalency Diploma.

Models adjusted for sex, age, race/ethnicity, household poverty, household educational attainment, insurance status, and household language.

*p<.05, **p<.01, ***p<.001

Table S4. Multivariable logistic regression of associations between behavioral/conduct problems and any disordered eating behaviors.

| **Characteristics** | **aOR (95% CI)** |
| --- | --- |
| **Behavior disorders** | 3.40 (3.03-3.81)*** |
| **Sex** |  |
| Male | REF |
| Female | 1.15 (1.07-1.23)*** |
| **Age (yrs.)** |  |
| 6-9 | REF |
| 10-12 | 0.96 (0.87-1.05) |
| 13-17 | 0.99 (0.92-1.07) |
| **Race/ethnicity** |  |
| Asian non-Hispanic | 0.78 (0.67-0.90)*** |
| Black non-Hispanic | 1.09 (0.97-1.22) |
| Hispanic | 1.18 (1.07-1.29)*** |
| Multi-racial/Other non-Hispanic | 1.11 (1.00-1.24) |
| White non-Hispanic | REF |
| **Household poverty** |  |
| < 100% FPL | 1.00 (0.88-1.13) |
| 100-199% FPL | 0.97 (0.87-1.08) |
| 200-399% FPL | 1.10 (1.01-1.19)* |
| ≥400% FPL | REF |
| **Household educational attainment** |  |
| Less than high school | 0.99 (0.82-1.21) |
| High school /GED | 1.07 (0.97-1.18) |
| Some college or technical school | 1.03 (0.95-1.13) |
| College degree or higher | REF |
| **Insurance status** |  |
| Private only | REF |
| Public only | 1.24 (1.12-1.37)*** |
| Public and private | 1.53 (1.32-1.77)*** |
| Currently uninsured | 1.02 (0.85-1.23) |
| **Household Language** |  |
| English | REF |
| Other than English | 1.06 (0.92-1.22) |

Source: National Survey of Children’s Health.

Abbreviations: aOR, adjusted odds ratio; CI, confident interval; FPL, federal poverty level (the ratio of total family income to the family poverty threshold); GED, General Equivalency Diploma.

Models adjusted for sex, age, race/ethnicity, household poverty, household educational attainment, insurance status, and household language.

*p<.05, **p<.01, ***p<.001

Table S5. Multivariable logistic regression of associations between attention-deficit/hyperactivity disorder and any disordered eating behaviors.

| **Characteristics** | **aOR (95% CI)** |
| --- | --- |
| **Attention-deficit/hyperactivity disorder** | 2.63 (2.41-2.88)*** |
| **Sex** |  |
| Male | REF |
| Female | 1.15 (1.08-1.23)*** |
| **Age (yrs.)** |  |
| 6-9 | REF |
| 10-12 | 0.91 (0.83-1.00) |
| 13-17 | 0.92 (0.85-0.99)* |
| **Race/ethnicity** |  |
| Asian non-Hispanic | 0.81 (0.70-0.94)** |
| Black non-Hispanic | 1.14 (1.02-1.26)* |
| Hispanic | 1.22 (1.11-1.34)*** |
| Multi-racial/Other non-Hispanic | 1.15 (1.03-1.27)* |
| White non-Hispanic | REF |
| **Household poverty** |  |
| < 100% FPL | 1.00 (0.88-1.13) |
| 100-199% FPL | 0.95 (0.86-1.06) |
| 200-399% FPL | 1.10 (1.02-1.19)* |
| ≥400% FPL | REF |
| **Household educational attainment** |  |
| Less than high school | 0.97 (0.80-1.18) |
| High school /GED | 1.06 (0.96-1.18) |
| Some college or technical school | 1.03 (0.95-1.12) |
| College degree or higher | REF |
| **Insurance status** |  |
| Private only | REF |
| Public only | 1.29 (1.17-1.42)*** |
| Public and private | 1.64 (1.41-1.89)*** |
| Currently uninsured | 1.07 (0.90-1.29) |
| **Household Language** |  |
| English | REF |
| Other than English | 1.04 (0.90-1.20) |

Source: National Survey of Children’s Health.

Abbreviations: aOR, adjusted odds ratio; CI, confident interval; FPL, federal poverty level (the ratio of total family income to the family poverty threshold); GED, General Equivalency Diploma.

Models adjusted for sex, age, race/ethnicity, household poverty, household educational attainment, insurance status, and household language.

*p<.05, **p<.01, ***p<.001
